# Machine learning-based Sr isoscape of southern Sardinia: A tool for bio-geographic studies at the Phoenician-Punic site of Nora

**DOI:** 10.1371/journal.pone.0287787

**Published:** 2023-07-19

**Authors:** Melania Gigante, Alessandro Mazzariol, Jacopo Bonetto, Elena Armaroli, Anna Cipriani, Federico Lugli

**Affiliations:** 1 Department of Cultural Heritage, University of Padua, Padua, Italy; 2 Department of Chemical and Geological Science, University of Modena & Reggio Emilia, Modena, Italy; 3 Lamont-Doherty Earth Observatory of Columbia University, Palisades, New York, United States of America; 4 Department of Cultural Heritage, University of Bologna, Ravenna, Italy; Austrian Academy of Sciences, AUSTRIA

## Abstract

Since prehistoric times, the island of Sardinia—in the western Mediterranean—has played a leading role in the dynamics of human population and mobility, in the circulation of raw materials and artefacts, idioms and customs, of technologies and ideas that have enriched the biological, linguistic and cultural heritage of local groups. For the Phoenician and Punic periods (from the 9^th^ to the 3^rd^ centuries BCE), the ancient site of Nora—in southern Sardinia—represents an emblematic case in the study of migratory phenomena that occurred on the Island from the Iron Age until the Roman conquest. Despite the importance of exploring (and characterising) such movements from a wider bio-cultural perspective, the application of bio-geochemical tools for geographical provenance to the ancient skeletal populations of Sardinia is yet scarce. The present work is the first step towards filling this gap with the development of the first isoscape of southern Sardinia using new bioavailable Sr isotope data and a machine-learning approach. From a geolithological point of view, Sardinia is rather heterogeneous and requires detailed studies to correctly assess the distribution of the isotopic signature of bioavailable Sr. The random forest model employed here to construct the Sr isoscape uses several external environmental and geological variables. The most important predictors are related to age and bedrock type, with additional input from local soil properties. A 10-fold cross-validation gives a mean square error of 0.0008 and an R-squared of 0.81, so the model correctly predicts the ^87^Sr/^86^Sr ratio of unknown areas. By using a Bayesian provenance assignment workflow, we tested the isoscape here produced to determine the geographic origin and the mobility of archaeological and modern fauna collected from the Phoenician-Punic site of Nora and the surrounding Pula Plain. Our results indicate that archaeological sheep and goats (^87^Sr/^86^Sr < 0.7090) are compatible with areas close to Nora and Pula Plain, in agreement with archaeological evidence of pastoralism in those areas. Modern wild and domesticated fauna (^87^Sr/^86^Sr > 0.7090) show compatibility with several natural and anthropogenic locations in southern Sardinia, as expected based on modern species distribution data. Finally, we discuss the large Sr isotopic variability of the Nora baseline, where human mobility studies of human cremated and inhumed individuals are currently underway.

## Introduction

Nowadays, the use of stable and radiogenic isotope analysis is widely exploited to detect and explore small and large-scale bio-geographical mobility at both individual and population levels. Isotope fingerprinting is applied to a variety of samples–e.g., biological tissues, artefacts, soils, and water–measuring various isotope ratios of elements such as oxygen, e.g., [1,2], hydrogen, e.g.,[3], lead, e.g., [4–6], sulphur, e.g.,[7], and strontium, e.g., [8–10].

The application of inorganic chemistry to mobility studies springs from the assumption that the characterization of the geographic origin or the movement of goods/people across the landscape is closely related to the geochemical signature of the different geographic localities. This signature is locked in water and soils and is largely related to the age of the local rocks and the mineralogical/chemical composition of the rock/substrate [11].

Strontium isotope ratios, in particular, are excellent tracers of low-temperature terrestrial processes due to the abundance of elemental Sr and its mobility between the bio-, geo-, and hydro-spheres [11]. In nature, strontium has four isotopes, i.e., ^84^Sr, ^86^Sr, ^87^Sr and ^88^Sr, of which only ^87^Sr is radiogenic, formed by the β- decay of rubidium ^87^Rb [11–13]. The strontium isotope signature (^87^Sr/^86^Sr) of a certain geological area depends on (i) the primary chemical composition of the rock, i.e., the Rb-Sr ratio at the time of crystallization; (ii) time passed since the closure of the system (crystallization). In general terms, a high Rb content combined with ‘old’ ages returns high ^87^Sr/^86^Sr ratios due to the radioactive decay of the long-lived ^87^Rb radionuclide to ^87^Sr stable isotope [14]. According to the large body of Sr isotope data available on geological formations, the Sr isotope signatures of certain categories of rock types can roughly vary as follow: (i) pure marine carbonates <0.710 (0.706–0.709) because of the interplay between hydrothermal and continental isotope contribution to the oceanic waters [15]; (ii) old granites, gneisses (>0.710) because of the high Rb concentration and radiogenic ingrowth; (iii) mantle-derived basalts <0.706 because of the low Rb abundance of the mantle source contributing to this magmatism; (iv) arc-basalts >0.706 because of the combined contribution of low Rb mantle and high Rb crust reservoirs [15,16].

Strontium passes from rocks to soil and groundwater through weathering processes, and from there to local vegetation and animals [8]. This Sr is the so-called *bioavailable strontium* that from the bedrock travels with water and soils through the hydrological and biological cycle and enters the food chain. Despite the number of physical processes (e.g., condensation, evaporation, diffusion) the ^87^Sr/^86^Sr ratio remains almost unchanged and undergoes a negligible fractionation because of the relatively small mass differences between strontium isotopes [16]. Hence, humans who primarily take up Sr through local food and water have ^87^Sr/^86^Sr similar to those of the local bedrock geology where the water has flown and where the produces were grown [8].

In the mammalian body, via its biochemical characteristics, elemental Sr replaces calcium, due to their equal bivalent state and similar ionic radius, during the development of (mineralized) tissues,–e.g., bone, dentine, enamel and cementum–transferring its ^87^Sr/^86^Sr isotope signature [17–20].

The interpretation of strontium isotope ratios for the provenance of archaeological materials requires comparison with a local map of the distribution of the ^87^Sr/^86^Sr ratio of the bioavailable Sr [21–24]. These local maps are generally described as ‘isoscape’ and can be built through different approaches [23]. The local bioavailable ^87^Sr/^86^Sr does not always follow the underlying geology, e.g.,[24–28], due to the mix of bedrock Sr with other Sr end-members (e.g., groundwater, precipitation, sea aerosol); therefore, measuring environmental archives that can integrate, at least in part, the local bioavailable Sr (e.g., plants with different root systems; soil leachates; ground and/or surface water; invertebrates such as snail shells and insects; domestic and wild non-migratory animals remains from modern and/or archaeological contexts) is crucial for a proper definition of the local bioavailable ^87^Sr/^86^Sr signature, e.g., [9,29,30].

An increasing number of country-regional-based bioavailable strontium isoscapes of different areas across Europe, the Mediterranean, South Africa, Asia and North-Central and South America, and Australia, are available in the literature. These maps have been constructed through various spatial interpolation and modelling approaches, e.g., [31–50]. For the Italian Peninsula, two different (isoscape) maps based on both new and published data have been recently proposed [51,52]. However, detailed analyses of the variations of bioavailable strontium isotope ratios across Italy on a macro- and micro-regional scale are lacking.

Here, we analysed plants, soil leachates, rocks, and (meteoric and ground-) waters to characterize the (bioavailable) Sr isotope variability in southern Sardinia. Being considered one of the most accurate environmental proxies for the reconstruction of the bioavailable Sr ratio, plants were then used to model the first strontium isoscape map for the sub-region of southern Sardinia, through a machine-learning approach. From a historical-archaeological perspective, the pivotal geographical position of this sub-region makes it of great interest for the study of mobility phenomena and cross-cultural interactions between foreigners and local populations across the central-western Mediterranean of the 1^st^ millennium BCE. Applied to provenance and mobility studies, the spatial distribution of bioavailable ^87^Sr/^86^Sr throughout southern Sardinia has the potential to enhance the interpretation of individual’s local, micro-regional or supra-regional movement patterns. This has been tested on Sr isotopes of modern and archaeological faunal skeletal remains from the site of Nora and its surroundings, by using an inverse Bayesian assignment method developed by Wunder [53]. The outputs of this approach are continuous-probability maps of geographic assignment, thus associating the estimation of individual provenance with a probability value, bypassing conventional ‘eyeballing’ attributions.

Finally, from a methodological point of view, the environmental features of the archaeological site of Nora highlight the importance of choosing appropriate samples when building the local isoscape and interpreting the local Sr isotope variability in provenance studies.

### Archeological significance of Sardinia

Sardinia is the second-largest island in the Mediterranean after Sicily. The Island is located in the middle of the western Mediterranean Sea, covering ∼24,000 km^2^ with a coastline of 1,849 km. A complex orographic pattern characterizes the Island with plain, hilly and mountainous landscapes placed on different geological substrate [54].

From an archaeological perspective–owing to its geographical location and the richness in metalliferous ore deposits–Sardinia has played since the Neolithic a key role in the human population dynamics, mobility, circulation of raw materials and artefacts, maritime networks and biocultural interactions in the Mediterranean, e.g., [55]. The presence on the island of an important obsidian source (the volcanic outcrops of Monte Arci in the Gulf of Oristano, western-central Sardinia) has undoubtedly encouraged the creation of regional and extra-insular networks which may have been followed by the movement of people [56]. As early as the Late Neolithic, obsidian originating from Sardinia is attested in several archaeological sites throughout the Mediterranean [57,58].

During the Nuragic period (∼1700–900 BCE), archaeological evidence suggests that Sardinia was at the centre of a wider trading network with the Italian Peninsula and the eastern Mediterranean [59–61]. Mycenean and Cypriot pottery have been identified in Nuragic contexts, such as at Nuraghe Antigori-Sarroch [62] and small numbers of eastern Mediterranean sherds have been found at sites such as Nuraghe Arrubiu-Orroli [63], Nuraghe Su Nuraxi-Barumini [64], and many others. Sardinia became a place of interactions between local people and Mycenaean, Euboean, Levantine and Cypriot merchants and sailors. These encounters are the prelude to the massive phenomena of cross-cultural interactions that occurred during the Phoenician expansion toward the Western Mediterranean and the so-called eight-century Greek colonization [65–68]. As for what has been attested in the Tyrrhenian regions of Southern Italy, it is only at the beginning of the Iron Age that transmarine contacts and human mobility have a new magnitude and significance. During the 9^th^-8^th^ centuries, up to the 7^th^-6^th^ centuries, BCE, Phoenicians, Greeks and Levantine traders and ‘colonists’ brought Sardinia, southern Italy and Sicily into a complex system of the political and economic network extending from Lebanon to the Iberian Peninsula’s coasts [69–73]. In particular, between 750 and 650 BCE and possibly earlier, Phoenicians established emporia and strongholds mainly (but not only) along the South and southwestern Sardinian coasts. Phoenician outposts (or *colonies*, from the 6^th^ to 3^rd^ century BCE [74], such as Cagliari, Sulcis and Nora not only became the major urban centres of Punic Sardinia but continued as important Roman cities.

#### The Phoenician and Punic site of Nora

The ancient site of Nora (*see* S1 Fig) is one of the most significant Phoenician foundations on the southern coast of Sardinia, mentioned by Pausanias as the oldest settlement on the Island (Paus. X, 17, 5) and established by the oikistes (the Greek founder of a colony) Norax from the Iberian Tartessus (Sol. IV, 2). The peculiar morphology of the peninsula on which Nora was founded, which finds close analogies with other Phoenician sites on the island, as well as in Sicily and the Levantine coasts [74], led to Nora being an essential stopover on the routes of Phoenician expansion and trade in the central and western Mediterranean from the 1^st^ millennium BCE onwards. Nora’s peninsula was among the few ports of call on the southern Sardinian coast to offer three good-weather coves for the makeshift shelter of vessels, as well as a safe harbour for the docking of ships located in the area of the present-day Peschiera [75,76].

Between 1997 and 2006, archaeological excavation campaigns conducted by the University of Padua beneath the Roman age forum allowed the reconstruction of the various cycles of obliteration and preparation of dwelling structures built in perishable materials from the end of the 7^th^ century BCE and, with increasing frequency, until the late second half of the following century [76]. However, the conspicuous amount of 8^th^ century BCE pottery in secondary deposits, suggests a more ancient frequentation of Capo di Pula (or *Pula Cape*, i.e., a promontory connected to the mainland by a sandbar; Pula Cape is situated West of Nora’s peninsula and is part of the Nora archaeological area) [77,78], confirmed by recent radiocarbon analyses of burnt plant remains [78].

Furthermore, the Roman temple from the Severian period yielded similar evidence from the stratigraphy underneath [79], reinforcing the interpretation of the early settlement of Nora as an outpost, changing in dimensions and shapes, sometimes enlarged and sometimes shrunken, continually obliterated and then renewed, in which goods and traders, Phoenicians, Etruscans, Greeks and local groups constantly moved around [80,81].

At Nora, two distinct funerary areas are located across the northern part of the peninsula, peripherally to the settlement. The two necropoles were used during the Phoenician period and, later on, with the Punic presence at the site. In particular, the north-western necropolis–where excavation campaigns are still ongoing–has yielded evidence of funerary rituals between the second quarter of the 7^th^ century BCE and the 3^rd^ century BCE [82,83]. Changes in the funerary customs and grave goods’ composition, the presence of imported items from the Italian Peninsula and the eastern Mediterranean and the use of exotic manufacturing techniques for certain ceramic forms (or parts of them), clearly indicate that Nora was a dynamic centre opened to external influxes; the Phoenician and Punic town was, indeed, a place where allochthonous populations took turns establishing complex dynamics of interrelation and exchange with the local Sardinian population [79,84].

### Geology of Sardinia

The island of Sardinia is geologically complex with outcropping lithologies varying between sedimentary, magmatic and metamorphic rocks ranging in age from the Cambrian to the Quaternary [85]. Sardinia is also rich in mineral deposits, which embrace a wide variety of types encompassing the geological evolution of the island and are related to extensive mobilization, migration, concentration and re-concentration of elements, such as Pb-Zn-Cu-Ag-F-Ba among the most important, to form different types of mineral deposits. From a tectonic point of view, Sardinia belongs to the so-called Sardinia-Corsica block, a fragment of the orogenic belt resulting from the collision of the Laurasia and Gondwana continents in the early Carboniferous, namely the Variscan or Hercynian orogeny. This Variscan basement consists of anchizonal to high-grade metamorphic rocks that were later intruded by Permo-Carboniferous granitoid [54,85]. The oldest rocks in the Sardinia outcrop in the southern portion are Early Cambrian sedimentary carbonates.

The evolution of Sardinia from the Permian to the Oligocene continues as a passive margin linked to the opening of the Thetys ocean with the deposition of a thick sedimentary cover characterized by marine limestones and terrigenous conglomerates. In the Oligocene-Miocene, concerning the tectonic rotation of the Sardinia-Corsica block due to the opening of the Balearic Sea, the deposition of a volcano-sedimentary succession characterized by rhyolitic-rhyolitic ignimbrites and basaltic-andesitic lava flows occurred around the basement at several locations [85]. After the Oligocene, the Sardinia block became a passive margin related to the opening of the Tyrrhenian Sea and sediment deposition in the form of carbonate-mixed siliciclastic successions that occurred in shallow marine and transitional environments. Late extensional tectonics during the Pliocene-Pleistocene in southern Sardinia produced large plateaux of intra-plate basalts of alkaline-sub alkaline affinity. More recent Quaternary deposits consist of continental, marine and a few aeolian deposits [85].

## Materials and methods

### Sample selection

**Fig 1:**
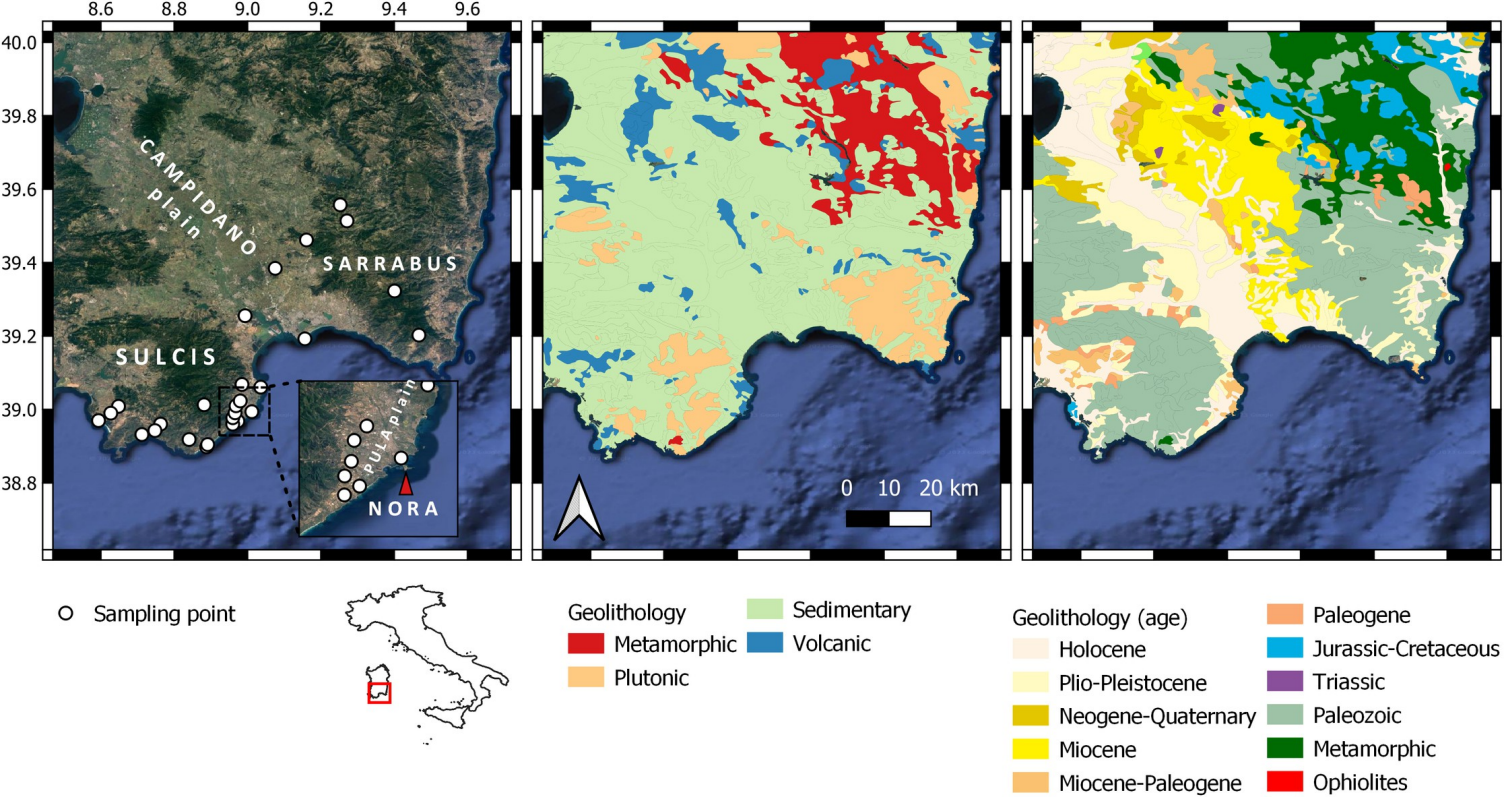
Geographical and geological framework of this study (Southern Sardinia). The left panel depicts the sampling spots, including the location of the Nora archaeological site and relevant places mentioned in the manuscript; the satellite map is provided by Google through the QGIS QuickMapServices plug-in. The central panel and the right panel report the geolithological features of the area, with the main formations classified by rock ‘type’ and age. These maps are based on the geolithological map of Italy available at the Geoportale Nazionale (http://wms.pcn.minambiente.it/ogc?map=/ms_ogc/wfs/Carta_geolitologica.map).

Fifty-two samples of bioapatite, plants, soils, rocks and water were collected from n = 27 sampling sites (Fig 1 and S1 Table), up to ∼65 km from the ancient site of Nora (38° 59’ 04" N; 9° 00’ 56" E). The sampling sites were carefully selected based on the underlying bedrock geology to cover the main geological features of the Southern Sardinia district. GPS coordinates were recorded for each sample site. In addition, to test the geo-biological Sr isotope variability at Nora, rock, soil and water samples were also collected at the site and its surroundings from areas with the same underlying geology.

Environmental, geological and biological samples (S1 Table) were classified by source and divided into five main classes, i.e., (i) *plant* (leaves of bushes and grasses grown on different lithological formations, e.g., andesites, granites, sandstone); (ii) (leachate) *soil* (-20 cm from the modern ground level; from archaeological stratigraphic levels at Nora and burial soil from Nora’s necropolis); (iii) *water* (meteoric and groundwater); (iv) *rock* (mainly outcrops); and (v) *mammal* (tooth enamel from archaeological and modern fauna, and bone from modern fauna).

Overall, both modern vegetation and faunal samples came from natural areas (i.e., low anthropic impact) to possibly reduce the contamination of fertilizers and pesticides that could introduce anthropogenic strontium [86]. Tooth enamel and bone samples from modern domesticated fauna here analysed (sheep-goat) were extracted from animals breeding in the (forested) areas surrounding the ancient site of Nora, e.g., the Pula Plain. These animals were grass-fed, without the use of processed food for animal consumption.

It should be noted that tooth enamel and bone samples from modern wild fauna (wild boar and deer) were collected from skeletonized animals. These remains were found in the wooden areas of the Pula Plain and its surrounding during plant and soil sampling activities for this study (*see also*, Additional Information and Competing Interest Statement) (Fig 1, *the left panel*).

Conversely, one of the two archaeological fauna samples (sheep) was part of the osteological assemblage pertaining to Inhumation T22 (US 1302) from Nora’s north-western necropolis [87]; whilst the second one comes from Phoenician-Punic stratigraphy below the Roman Temple in Nora’s urban centre [88] (*see also*, S1 Table).

### Sr isotope analyses

Bone and tooth enamel samples were cleaned with MilliQ water and digested using concentrated suprapur HNO_3_. The bioavailable Sr fraction from soils was extracted using 0.25 M acetic acid [29,89]. Bulk rock samples were digested in closed-PTFE vessels on the hotplate (150°C), using a mixture of concentrated HNO_3_, HCl and HF, until total dissolution. Waters were filtered (5 μm) and acidified with HNO_3_ to a concentration of 3 M [90]. Plant samples were air-dried, ashed at 650° C and digested with concentrated suprapur HNO_3_ [91]. All samples but the waters were dried down and re-dissolved in 3M HNO_3_.

Strontium was purified in 30 μL-volume columns filled with Eichrom Sr spec–resin (100–150 μm bead size) as described in [90]. Sr was eluted with MilliQ water and collected in clean polypropylene vials. Each solution was then adjusted to 4% w/w HNO_3_ for MC–ICPMS analysis. The whole lab procedure was performed in the clean room of the Geochemistry Lab of the Department of Chemical and Geological Science at the University of Modena & Reggio Emilia, where the total lab blank is <100 pg.

The ^87^Sr/^86^Sr ratio of the samples was determined using a Neptune MC–ICPMS (Thermo Fisher Scientific) housed at the Centro Interdipartimentale Grandi Strumenti (CIGS) of the University of Modena & Reggio Emilia, as described in [91–94]. Sr solutions were diluted to ∼50 ppb and introduced through an APEX desolvating system. Masses (m/z) ^82^Kr, ^83^Kr and ^85^Rb were measured to correct for isobaric interferences. Mass bias normalization was performed by using an ^88^Sr/^86^Sr ratio of 8.375209 [95] and an exponential law. Repeated analyses of the NIST-SRM987 yielded an ^87^Sr/^86^Sr ratio of 0.710241 ± 0.000023 (2 SD, n = 30). Samples were reported to an accepted NIST-SRM987 value of 0.710248 [15].

#### Geospatial modelling

To build the local isoscape we relied on the plant samples (n = 30, from n = 27 locations) because they are currently considered one of the most accurate environmental proxies for the reconstruction of the bioavailable Sr isotope signature [96]. Concerning the plant samples from the site of Nora (n = 4), we calculated and used a median value. Data were imported in R (v. 4.0.5) and modelled by using the machine-learning method outlined in Bataille et al. [97]. Specifically, we employed a Random Forest (RF) algorithm based on multiple external predictors to model the isotope ratio at a 1 km resolution.

Several global raster maps (n = 21 from Bataille et al. [97]) of environmental and geological features were resampled at the locations where specimens were collected. The labelled dataset obtained after the resampling was employed to train the RF model, and then applied spatially to the area of interest. N = 6 variables were selected by *VSURF* based on their importance as external predictors, i.e., *r*.*srsrq1* is the predicted first quartile of the global ^87^Sr/^86^Sr model reported in Bataille et al. [98]; *r*.*bulk* is the soil bulk density (kg/m^3^); *r*.*clay* is the clay soil content (weight %); *r*.*cec* is the soil cation exchange capacity; *r*.*minage_geol* is the log of the minimum geological age from GLiM (high-resolution Global Lithological Map; [99]); *r*.*fert* is the global nitrogen fertilization. Random trees were thus built with n = 2 random variables at a time (i.e., optimized *mtry* parameter = 2).

To obtain a spatial-uncertainty map, we employed a quantile RF regression (*raster* package), then halving the RF q_0.84_—q_0.16_ difference (i.e., lower and upper of a ∼68% interval; [45]). The root-mean-squared error (RMSE) of a 10-fold cross-validation with n = 5 repetitions was used to test the accuracy of the model prediction. The importance of the predictors in the random forest model is computed based on two factors: the *%IncMSE* which is the relative increase of the cross-validation mean squared error, randomly permuting values of that specific variable and the *IncNodePurity* which expresses how much a specific variable impacts the tree-split.

#### Fauna provenance

Bones and teeth specimens collected from archaeological and modern fauna were tested for their provenance by using a Bayesian assignment method against the South Sardinia isoscape. The statistical analysis used the R package *assignR* by Ma et al. [100]. The isotope ratio of each sample was compared probabilistically to the isoscape and its associated prediction error, by using a Bayesian inversion method [53]. The *a priori* assumption is that the sample can come equally from each cell of the isoscape. The posterior probability of sample origin is computed at each grid cell, returning a raster object which contains one probability density surface per sample with its likely provenance. Finally, each raster map was normalized to its maximum value to force the scale between 0 and 1, where → 0 indicates that the sample is unlikely to come from that cell, while → 1 suggests that the sample is likely to come from that cell.

## Results and discussion

### Data description

**Fig 2:**
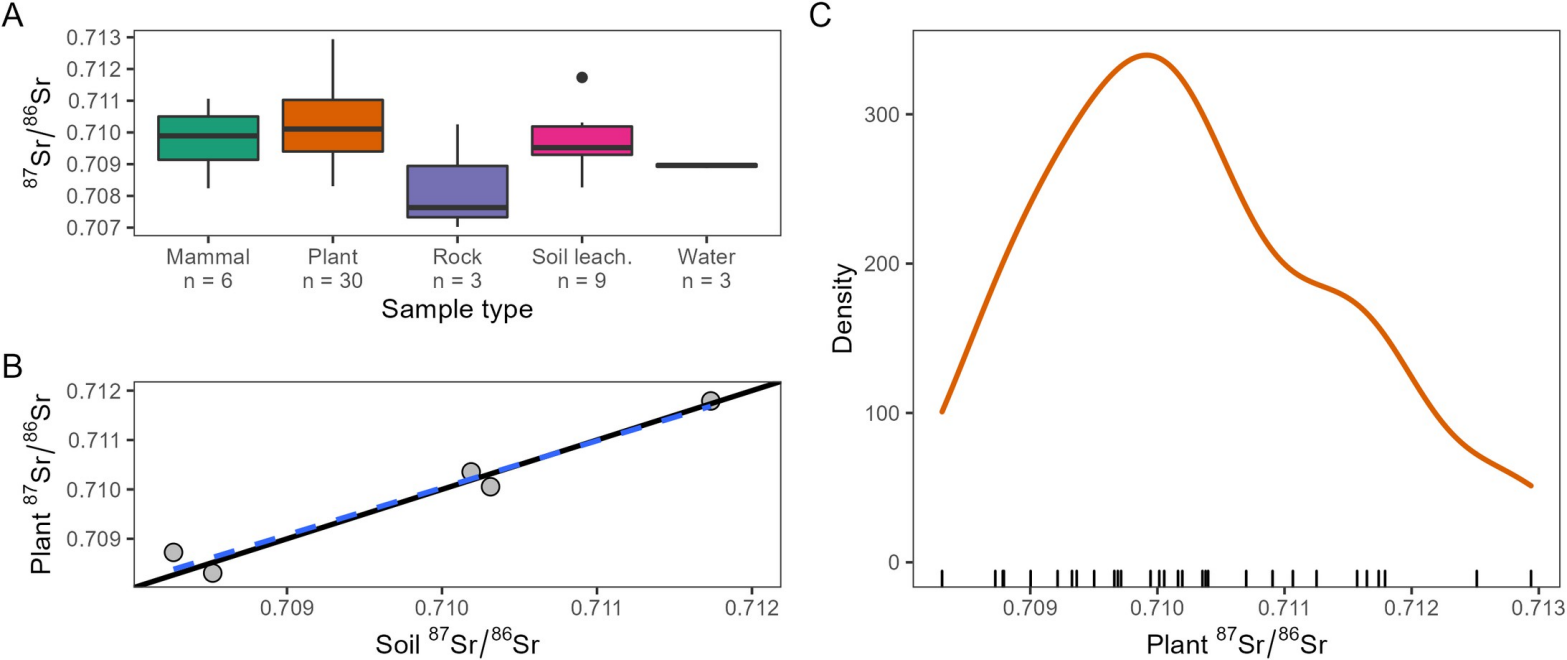
Sr isotope results. (A) Box plot graph illustrating the Sr isotope results of the environmental samples (i.e., mammals, plants, rocks, leachate soils, and waters) analysed in this study. (B) Comparison of plants and soil leachates Sr isotopes by five sample sites; the solid line is a 1:1 ratio; the blue dashed line is a linear regression fit. (C) Kernel density estimation of Sr isotope data obtained for plants analysed in this study.

Overall, the ^87^Sr/^86^Sr ratios of the samples range between 0.85613 (granite) and 0.70702 (andesite). Considering the bioavailable specimens only (i.e., excluding rocks) the Sr isotope ratio varies between 0.70824 (soil leachate) and 0.71294 (plant). Summary isotope data for each sample type are reported in Fig 2 and Table 1 (*please note* that the granite sample was ignored to improve data readability).

**Table 1:** Descriptive statistics of the Sr isotope data measured in this study. Summary isotope data for each sample type (i.e., mammal, plant, rock, soil leachate, and water). For each sample category, the main statistic parameters are provided.

Sample type	Mean	SD	Min	Max	Median	n
Mammal	0.70978	0.00105	0.70824	0.71106	0.70989	6
Plant	0.71026	0.00116	0.70831	0.71294	0.71011	30
Rock*	0.70830	0.00172	0.70702	0.71026	0.70763	3
Soil leachate	0.70964	0.00103	0.70827	0.71173	0.70952	9
Water	0.70896	0.00007	0.70888	0.70902	0.70896	3

*Granite sample excluded, ^87^Sr/^86^Sr = 0.85613.

^87^Sr/^86^Sr ratios of plant and soil samples collected at the same five locations return an elevated coefficient of determination (R^2^ = 0.94, p = 0.003) and an intercept close to 1 (0.95 ± 0.11). This suggests that, as expected, the shallow-rooted plants here considered mainly incorporated the bioavailable strontium pool of the (top)soil-leachable portions [44].

As reported in Materials and methods (see *Sample Selection*), plants only were used to model the local isoscape (mean = 0.71026, median = 0.71011, SD = 0.00116, n = 30). Plant data show a close-to-normal distribution (Kolmogorov-Smirnov test p-value = 0.75) with a skewness of 0.50 and a kurtosis of -0.31.

### Isoscape

**Fig 3:**
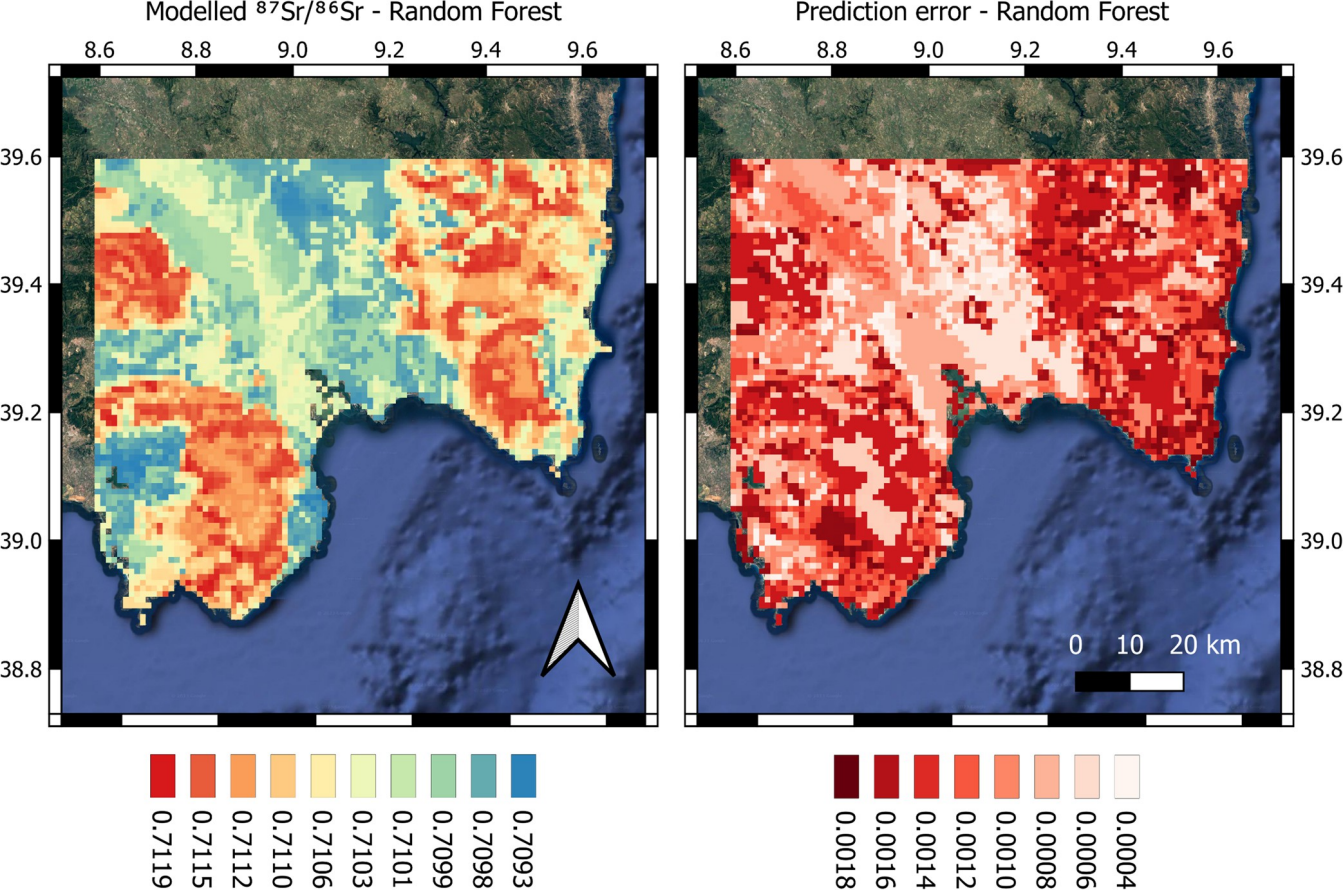
Sr-isotope ratios predicted by using a Random Forest algorithm. *Left panel*, modelled ^87^Sr/^86^Sr isotope ratio of South Sardinia by using a Random Forest algorithm with n = 5 external predictors. The 10-fold cross-validation resulted in RMSE = 0.0008 and R^2^ = 0.81; *right panel*, associated spatial uncertainties were obtained from a quantile Random Forest regression (*ranger* R package) and calculated as half of the (quantiles) q_0.84_—q_0.15_ difference. Both maps are given with a spatial resolution of 1 km.

The obtained 1 km-resolution RF isoscape map is illustrated in Fig 3. The southern Sardinia isoscape maps can be freely downloaded as a GeoTIFF from https://www.geochem.unimore.it/sr-isoscape-of-italy/ and S2 and S3 Figs of this article.

**Fig 4:**
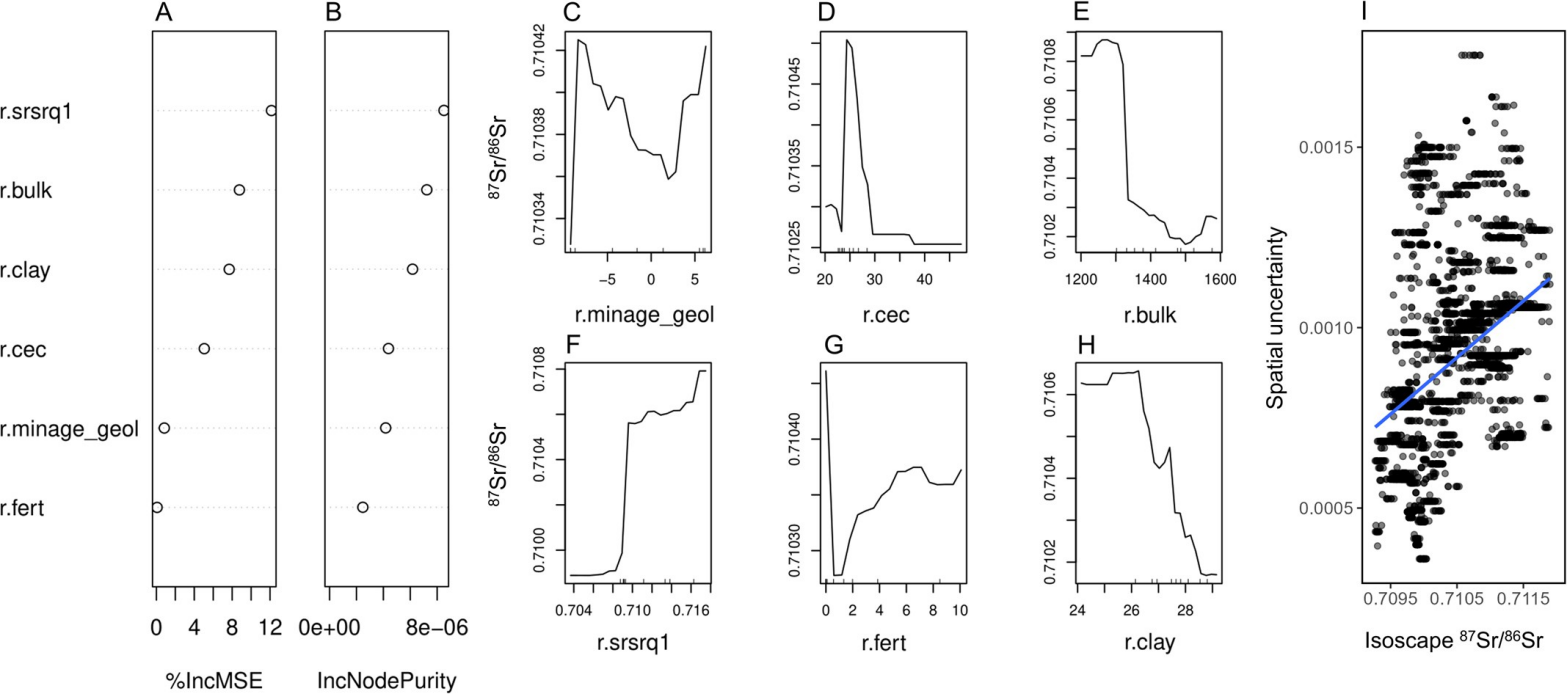
Performance of the Random Forest model. (A, B) Variable importance for the Random Forest model used in this work. The six variables (*r*.*srsrq1*, *r*.*bulk*, *r*.*clay*, *r*.*cec*, *r*.*minage_geol* and *r*.*fert*) were selected by using the *VSURF* algorithm. *See also* Bataille et al. [97]. The ‘%IncMSE’ (A) represents the relative increase of the cross-validation mean squared error, randomly permuting values of that specific variable (the higher the value, the more important the variable is in the model prediction). The ‘IncNodePurity’ (B) expresses how much a specific variable impacts the tree-split; (C, H) partial dependence plot for the VSURF-selected variables, showing their nonlinear behaviour: *r*.*srsrq1* is the predicted first quartile of the global ^87^Sr/^86^Sr model reported in Bataille et al. [98]; *r*.*bulk* is the soil bulk density (kg/m^3^); *r*.*clay* is the clay soil content (weight %); *r*.*cec* is the soil cation exchange capacity; *r*.*minage_geol* is the log of the minimum geological age from GLiM; *r*.*fert* is the global nitrogen fertilization. (I) Values predicted by the RF model (isoscape ^87^Sr/^86^Sr) plotted vs. their respective spatial uncertainties obtained through the quantile RF; the blue line is a linear regression fit (R^2^ = 0.17, p < 0.01).

The modelled Sr isotope values range between 0.70927 and 0.71190, highlighting a slight overestimation of the low-^87^Sr/^86^Sr in the modelled dataset compared to the measured samples (n = 4 plant samples are lower than 0.7091). The 10-fold cross-validation (i.e., partitioning of the dataset into multiple subsets to evaluate model performance) results in RMSE = 0.0008 and 81% of the variance explained, indicating that the algorithm properly predicts Sr isotope values of unknown areas. As suggested by the RF model, some specific environmental variables seem to better predict the Sr isotope ratios than others (Fig 4).

The bedrock model of Bataille et al. [98] (i.e., *r*.*srsrq1*) is one of the predominant predictors for the isoscape. This model calculates the ^87^Sr/^86^Sr ratio of the local bedrock based on the Rb content, the lithology and the age of the substrate using global maps (*see also* [38]) and it is particularly useful for those areas with a large geolithological heterogeneity, such as Southern Sardinia. Similarly, the log of the geological age from GLiM (*r*.*minage_geol*) contributed to the prediction, although with a little %IncMSE (*see* Fig 4, *panels C*, *H*), compared to *r*.*srsrq1*. In addition, soil properties (*r*.*cec*, *r*.*bulk* and *r*.*clay*) were selected as predictors of the bioavailable Sr isotopes, being soil one of the main bioavailable Sr reservoirs. The bioavailable Sr isotope ratio decreases e.g., with clay content, possibly indicating the relevant contribution of carbonate weathering in high-clastic soils [97]. Notably, no variables related to the sea spray seem to have largely influenced the model prediction (not selected by *VSURF*), suggesting that ocean-derived strontium had a limited effect on the bioavailable Sr of Southern Sardinia [101]. On the other hand, a remarkably small effect of nitrogen fertilizer (*r*.*fert*) may suggest a very limited or no contribution of anthropic practices to the Sr isotope regional values [86]. Overall, the highest radiogenic Sr values (> ∼0.711) mainly correspond to the old Paleozoic areas of the region, while the lowest to the young Holocene-Pliocene sedimentary units.

The Sr isoscape is associated with a spatial-uncertainty map (ranging between 0.00036 and 0.00175), obtained with a quantile RF model. The highest prediction errors of the map (up to ∼0.0018) are localized in the most radiogenic areas (*see* Fig 4), as found e.g., by Bataille et al. [38] for their Europe isoscape. The most likely explanation is that more radiogenic values are linked to old metamorphic and igneous areas where the bedrock isotope signatures tend to be remarkably different from those of other mixing less radiogenic end-members (e.g., aerosol, seawater, precipitation), hampering the model predictions.

### The bioavailable Sr isotope baseline at the Nora peninsula

Southern Sardinia shows a remarkable heterogeneity in terms of geolithology and, in turn, in Sr isotope ratios, hampering the definition of a local baseline for Nora. The plant samples collected at the site itself display indeed a large isotopic variability (range = 0.70878–0.70969; max-min = 0.00090; median = 0.70942), at less than 500 m distance. This is mainly due to the presence within the site of several different Sr end-members, with different Sr isotope signatures, namely meteoric water (∼ 0.7090, also similar to other values for Italy [52]), seawater (∼ 0.7092), volcanic rocks (∼ 0.7070–0.7076), and clastic sedimentary rocks (∼ 0.7102). Altogether, the bioavailable samples (e.g., waters, plants and soil leachates) measured at the site provided a median value of 0.70933 and an interquartile range (IQR) of 0.00052.

**Fig 5:**
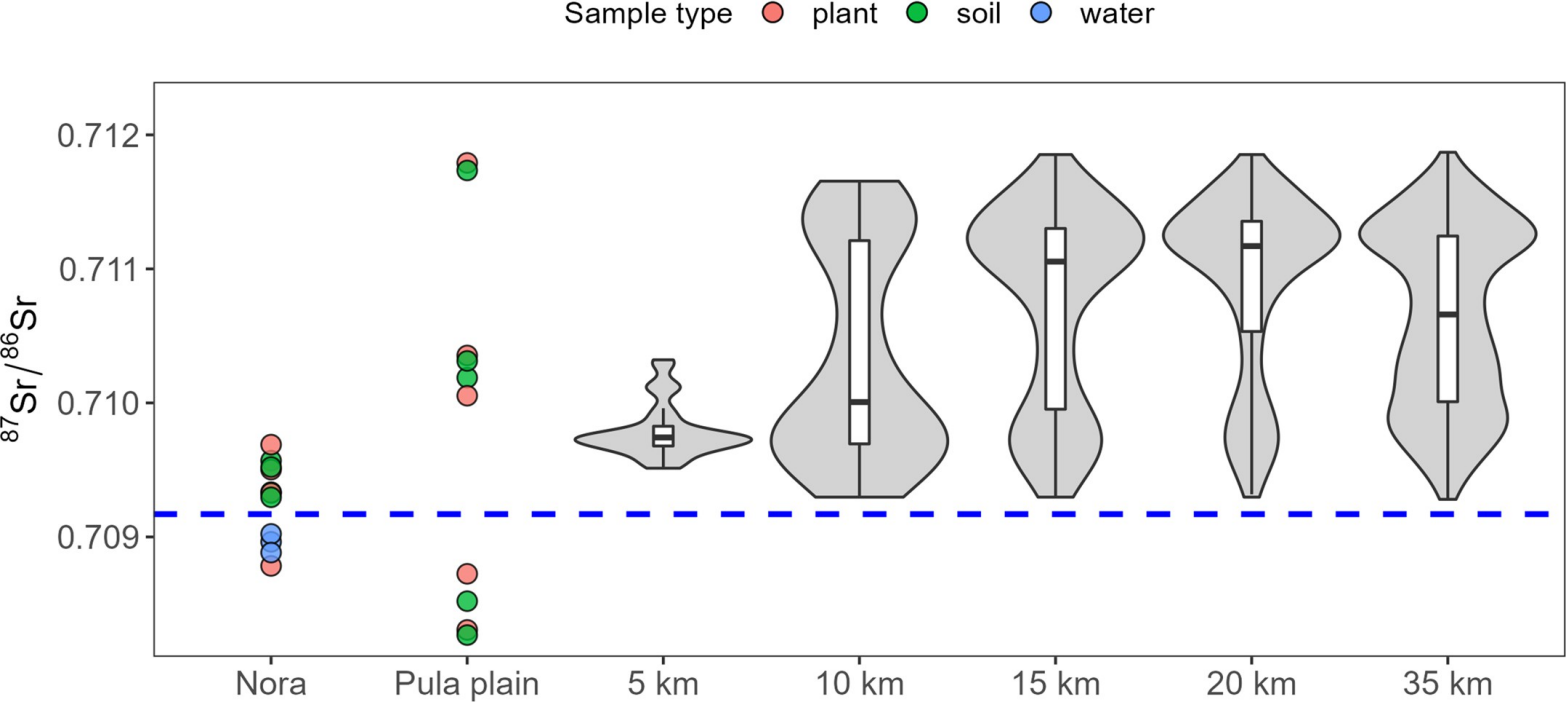
Sr isotope baseline at Nora and surroundings (i.e., Pula Plain, up to 35 km from the Nora site). Coloured dots are environmental samples from Nora and the Pula Plain. Violin plots represent the Sr isotope distributions of the RF isoscape, extrapolated at different radial distances from Nora. The blue dashed line represents the Sr isotope composition of modern seawater (0.70917).

By extrapolating Sr values of the isoscape cells at different radial distances from the site (Fig 5), it is evident that at 5 km from the site—which approximately corresponds to the Pula Plain–the Sr range is relatively narrow (IQR = 0.00015; max-min = 0.0008)–, but it increases already at 10 km (IQR = 0.00152; max-min = 0.00236), reflecting almost the entire variance registered in the isoscape.

Similarly, soil and plant samples collected at and around the site (including the Pula Plain) range from 0.70827 to 0.71179 (Fig 5), indicating a large bioavailable Sr isotope variability linked with the geological heterogeneity of the area.

### Isotopic provenance assignment of fauna samples

**Fig 6:**
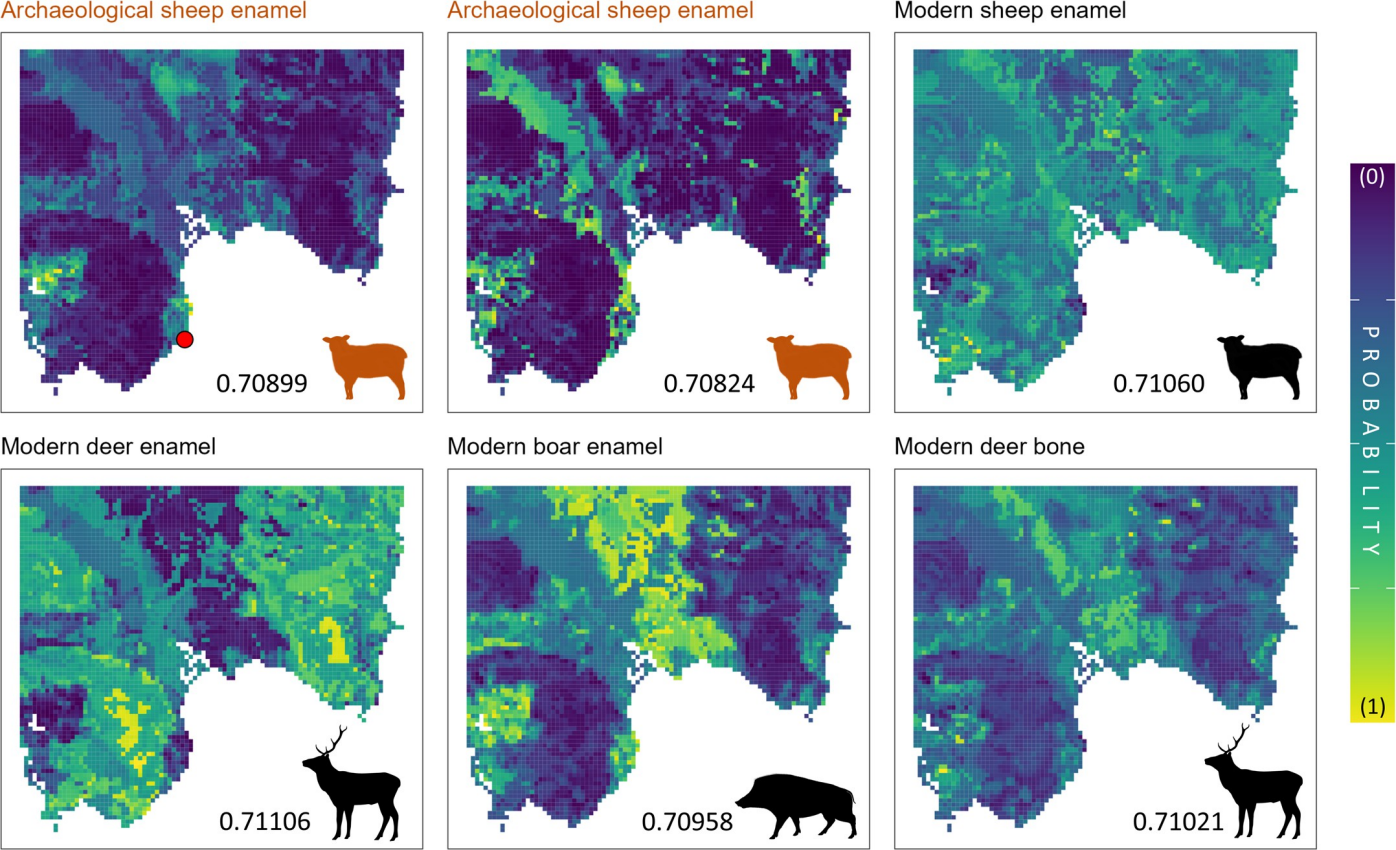
Bayesian probability provenance assignment of the six fauna samples analysed in this study. Modern (n = 4; in Fig 6, black silhouette) and archaeological (n = 2; in Fig 6, dark orange silhouette) samples collected at the Nora site and surroundings. The red dot in the first panel depicts the location of the Nora site. The yellowish areas represent those with a higher probability of origin. Animal silhouettes are from phylopic.org.

The probabilistic provenance assignment of n = 6 fauna samples (Fig 6) has been tested based on the southern Sardinia isoscape.

The two archaeological sheep enamel samples provided Sr isotope values of 0.70899 and 0.70824, fitting the areas close to the site (i.e., Pula Plain; Fig 1). It is worth noting that such low radiogenic values are absent from the isoscape alone, whose minimum ^87^Sr/^86^Sr ratio is 0.70927; however, they still correspond to some areas of the map owing to the contribution of the spatial uncertainty. In addition, some vegetation, water and soil leachate samples from Nora and the Pula Plain show ratios < 0.7090, thus compatible with the sheep values. Nevertheless, the isotope matching of the ancient sheep with the Pula Plain finds agreement with the archaeological evidence and the known practice of sheep herding in the area [102,103].

Modern animals display higher Sr isotope ratios (> 0.7095) than archaeological sheep. This suggests that they dwelled in different geological areas compared to the sheep found at the Nora site. Interestingly, the deer enamel sample (0.71106) indicates that the individual likely comes from the Sulcis or the Sarrabus territories (southwestern and southeastern portions of the map respectively; (Fig 1), which are known as the main forested areas, where modern Corsican red deer are distributed [104]. Yet, the deer bone shows a slightly less radiogenic value (0.71021) more compatible with the Campidano Plain (*see* Fig 1), between the Sulcis and the Sarrabus mountain areas, but also with some spots located in the western Sulcis. These areas are however not or little suitable as red deer habitats [104], mainly due to their relatively heavier urbanization.

Hence, the isotope matching between the deer bone and the plain can be explained as due to the mix of different high and low radiogenic Sr end-members in the bone reservoir of the individual, fixed during several years of adult life. This suggests in turn either a large home range for the adult deer individuals or the use of different environmental sources not reflected in our isoscape. Akin to the modern deer bone, the enamel specimen collected from the modern boar shows an isotope ratio (0.70958) compatible with the Campidano Plain and western Sulcis, but also with the Pula Plain. Wild boars in Sardinia are ubiquitous and occupy different habitats including anthropic areas [105], as those highlighted by the provenance assignment map. The individual considered in our study was found skeletonized in the Pula Plain, thus agreeing with its Sr isotopic signal. The modern sheep enamel shows an ^87^Sr/^86^Sr ratio of 0.71060 between those of the boar and the deer enamel. This value is compatible with many areas of the Southern Sardinia landscape including the Campidano Plain, the Sulcis mountains and part of the Pula Plain. Akin to the wild boar, this sheep was naturally bred in the Pula Plain and surroundings, thus consistent with the isotope fingerprinting.

## Conclusions

Due to the great geological heterogeneity of southern Sardinia, the Sr isotope signature displays a large variability, thus hampering the study of human mobility through the analysis of the bioavailable Sr without an adequate investigation of the local baseline. Therefore, we report in this article novel environmental Sr isotope data and the first isoscape of southern Sardinia, modelled using a machine learning algorithm.

The work presented here is of twofold importance. Firstly, it represents the first attempt to build an isoscape of the strontium bioavailable isotope ratio for Sardinia, which will enhance the current isoscape mapping for the Italian island regions. From a historical-archaeological perspective, this work constitutes a significant step towards understanding the dynamics of peopling and bio-geographic mobility in ancient Sardinia. The definition of a local strontium bioavailable baseline for the site of Nora is crucial in reconstructing and characterizing the influx of foreigners to the site during the Phoenician and Punic phases of peopling of Nora’s peninsula, as part of an ongoing research project.

The strontium isotope model produced here will be employed and compared with other baselines available in the literature for Mediterranean regions which ancient written sources and/or archaeological evidence have suggested to be a potential place of origin of individuals who arrived at Nora or with whom Nora’s inhabitants traded (e.g., Tyrrhenian coasts, Etruscan-Latium region, southern Spain and Balearic Islands, northern Africa).

Moreover, this model has proven successful and can be effectively used to study sub-regional human and faunal movements. This has been demonstrated by successfully assign the geographical provenance of modern and archaeological faunal samples (sheep, deer and boar) based on a Bayesian method. Our assignments are consistent with the modern known ecology of the animals and historical sources, supporting the use of the isoscape as provenance tool. Hence, the map can be exploited to investigate intra-insular mobility, which includes the movements of individuals from-and-to Nora and other settlements in the inland and coastal areas of southern Sardinia, involved in Phoenician-Punic and local settlement and occupation phenomena.

The widespread use of (multi)isoscapes, combined with a probabilistic assignment of origin, can overcome the current limitations of the canonical dualistic ’local/non-local’ approach, which assigns a specific probability of origin to the individual. To this end, it is necessary to improve the delineation of isoscape maps (e.g. with new statistical methods) and to increase the number of studies on the local distribution of bioavailable Sr isotopes.

## Supporting information

S1 Fig The ancient site of Nora (Cagliari, South Sardinia). On the left is the archaeological site of Nora. On the right are some ancient monuments of the Imperial Roman period (a., Terme a mare–Bath by the sea; b., Tempio Romano–Roman Temple; c., Casa dell’Atrio tetrastilo–House of the Tetrasyle Atrium. These images are the propriety of the Department of Cultural Heritage–at the University of Padua. The photographer Gianni Alvito (Teravista, CA) provided us with courtesy of the Ministero della Cultura–Italian Ministry for Culture (former Ministero per i Beni e le Attività Culturali e il Turismo), Soprintendenza Archeologia, Belle Arti e Paesaggio per l’area metropolitana di Cagliari e le province di Oristano e Sud Sardegna.
(TIF)

S2 Fig Southern Sardinia Isoscape as GEOTIFF. (TIF)

S3 Fig Spatial uncertainty of the Southern Sardinia Isoscape as GEOTIFF. (TIF)

S1 Table Sr isotope results of the samples considered in this study. List of the environmental samples analysed in this work and the ^87^Sr/^86^Sr isotope ratio results for each sample.
(DOCX)

S1 File (DOCX)

## References

[1] Pellegrini M., Pouncett J., Jay M., Pearson M.P., Richards M.P., 2016. Tooth enamel oxygen ‘isoscapes’ show a high degree of human mobility in prehistoric Britain. Scientific Reports. 6, 34986. doi: 10.1038/srep34986 27713538 PMC5054518

[2] Pederzani S., Britton K., 2019. Oxygen isotopes in bioarchaeology: principles and applications, challenges and opportunities. Earth-Science Reviews. 188, 77–107.

[3] Soto D.X., Wassenaar L.I., Hobson K.A., 2013. Stable hydrogen and oxygen isotopes in aquatic food webs are tracers of diet and provenance. Functional Ecology. 27, 535–543.

[4] Vautour G., Poirier A., Widory D., 2015. Tracking mobility using human hair: what can we learn from lead and strontium isotopes? Science & Justice. 55, 63–71. doi: 10.1016/j.scijus.2014.10.001 25577009

[5] Smith K.E., Weis D., Amini M., Shiel A.E., Lai M., Gordon K., 2019. Honey as a biomonitor for a changing world. Nature Sustainability. 2, 223–232.

[6] Killick D.J., Stephens J.A., Fenn T.R., 2020. Geological constraints on the use of lead isotopes for provenance in archaeometallurgy. Archaeometry. 62, 86–105.

[7] Bataille et al. 2021. Triple sulfur-oxygen-strontium isotopes probabilistic geographic assignment of archaeological remains using a novel sulfur isoscape of western Europe. PloS One. 16(5), e0250383. doi: 10.1371/journal.pone.0250383 33951062 PMC8099095

[8] Prince T.D., Burton J.H., Bentley R.A., 2002. The characterization of biologically available strontium isotope ratios for the study of prehistoric migration. Archaeometry. 44, 117–135.

[9] Evans J.A., Montgomery J., Wildman G., 2009. Isotope domain mapping of ^87^Sr/^86^Sr biosphere variation on the Isle of Skye, Scotland. Journal of the Geological Society of London. 166, 617–631.

[10] Lugli et al. 2019. Strontium and stable isotope evidence of human mobility strategies across the last Glacial Maximum in southern Italy. Nature Ecology and Evolution. 3, 905–911. doi: 10.1038/s41559-019-0900-8 31086279

[11] Bentley R.A., Price T.D., Stephan E., 2004. Determining the ‘local’ 87Sr/86Sr range for archaeological skeleton: a case study from Neolithic Europe. Journal of Archaeological Science. 31, 365–375.

[12] Kutschera W., Müller W., 2003. ‘Isotope language’ of the Alpine Iceman investigated with AMS and MS. Nuclear Instruments and Methods in Physics Research Section B: Beam Interactions with Materials and Atoms. 204, 704–719.

[13] Faure G., Mensing T.M., 2005. Isotopes: Principles and Applications, third ed. John Wiley & Sons, Hoboken, New Jersey (2005).

[14] Dickin A.P., 1995. Radiogenic Isotope Geology. Cambridge University Press, Cambridge.

[15] McArthur J.M., Howarth R.J., Bailey T.R., 2001. Strontium isotope stratigraphy: LOWESS version 3: best fit to the marine Sr-isotope curve for 0–509 Ma and accompanying look-up table for deriving numerical age. Journal of Geology. 109(2), 155–170.

[16] Capo R.S., Stewart B.W., Chadwick O.A., 1998. Strontium isotopes as tracer of ecosystem processes: theory and methods. Geoderma. 82, 197–225.

[17] Hillson S., 2005. Teeth. Cambridge University Press, Cambridge.

[18] AlQahtani S.J., Liversidge H.M., Hector M.P., 2010. Atlas of tooth development and eruption. American Journal of Physical Anthropology. 142(3), 481–490. 20310064 10.1002/ajpa.21258

[19] Beaumont J., Montgomery J., 2015. Oral histories: a simple method of assigning chronological age to isotopic values from human dentine collagen. Annals of Human Biology. 42, 407–414. doi: 10.3109/03014460.2015.1045027 26225904

[20] Frei et al. 2015. Tracing the dynamic life history of a Bronze Age Female. Scientific Reports. 5(1), p. 10431. 25994525 10.1038/srep10431 PMC4440039

[21] Bowen et al. 2009. Isoscape to address large-scale Earth science challenges. Eos Transitions American Geophysical Union. 90(3), 109–110.

[22] Bowen G., 2010. Isoscapes: spatial patterns in isotopic biogeochemistry. Annual Review of Earth and Planetary Sciences. 38, 161–187.

[23] Holt E., Evans J.A., Madgwick R., 2021. Strontium (87Sr/86Sr) mapping: a critical review of methods and approaches. Earth-Science Reviews. 103593.

[24] Beard B.L., Johnson C.M., 2000. Strontium isotope composition of skeletal material can determine the birthplace and geographic mobility of humans and animals. Journal of Forensic Science. 45(5), 1049–1061. 11005180

[25] Hamilton M., Nelson S.V., Fernandez D.P., Hunt K.D., 2019. Detecting riparian habits preferences in ‘savanna’ chimpanzees and associated fauna with strontium isotope ratios: implications for reconstructing habitat use by chimpanzee-human last common ancestor. American Journal of Physical Anthropology. 170(4), 551–556. 31633810 10.1002/ajpa.23932

[26] Frei K.M., Frei R., 2013. The geographic distribution of Sr isotopes from surface waters and soil extracts over the island of Bornholm (Denmark)–A base for provenance studies in archaeology and agriculture. Applied Geochemistry. 38, 147–160.

[27] Adams et al. 2019. A Strontium isoscape of north-east Australia for human provenance and repatriation. Geoarchaeology. 34, 231–251.

[28] Britton et al. 2020. Sampling plants and malacofauna in 87Sr/86Sr Bioavailability Studies: implication for isoscape mapping and reconstructing of past mobility patterns. Frontiers in Ecology and Evolution. 8, 579173.

[29] Esposito C., et al. 2023. Intense community dynamics in the pre-Roman frontier site of Fermo (ninth-fifth century BCE, Marche, central Italy) inferred from isotopic data. Scientific Reports. 13(1), 3632. 36869081 10.1038/s41598-023-29466-3 PMC9984403

[30] Montgomery J., Evans J.A., Wildman G., 2006. ^87^Sr/^86^Sr isotope composition of bottled British mineral waters for environmental and forensic purposes. Applied Geochemistry. 21, 1626–1634.

[31] Evans J.A., Montgomery J., Wildman G., Boulton N., 2010. Spatial variations in biosphere ^87^Sr/^86^Sr in Britain. Journal of the Geological Society of London. 167, 1–4.

[32] Frei K.M., Frei R., 2011. The geographic distribution of strontium isotopes in Danish surface waters–A base for provenance studies in archaeology, hydrology and agriculture. Applied Geochemistry. 26, 326–340.

[33] Bataille C.P., Bowen G.J., 2012. Mapping ^87^Sr/^86^Sr variations in bedrock and water for large-scale provenance studies. Chemical Geology. 304, 39–52.

[34] Pestle W.J., Simonetti A., Curet L.A., ^87^Sr/^86^Sr variability in Puerto Rico: geological complexity and the study of paleomobility. Journal of Archaeological Science. 40, 2561–2569.

[35] Hartman G., Richards M., 2014. Mapping and defining sources of variability in bioavailable strontium isotope ratios in the Eastern Mediterranean. Geochimica et Cosmochimica Acta. 126, 250–264.

[36] Copeland et al. 2016. Strontium isotope investigation of ungulate movement patterns on the Pleistocene Paleo-Agulhas plain of the Greater Cape floristic region, South Africa. Quaternary Science Review. 141, 65, 84.

[37] Laffoon J.E., Sonnemann T.F., Shafie T., Hofman C.L., Brandes U., Davies G.R., 2017. Investigating human geographic origins using dual-isotope (87Sr/86Sr, δ18O) assignment approaches. PloS One. 12, e0172562. 10.1371/journal.pone.0172562 PMC5319690 28222163

[38] Bataille C.P, von Holstein J.E., Laffoon M., Willmes M., Liu X.M., Davies G.R., 2018. A bioavailable strontium isoscape for Western Europe: a machine learning approach. PloS One. 13. e0197386. 10.1371/journal.pone.0197386 PMC5976198 29847595

[39] Willmes et al. 2018. Mapping of bioavailable strontium isotope ratios in France for archaeological provenance studies. Applied Geochemistry. 90, 75–86.

[40] Ladegaard-Pedersen P., Achilleos M., Dörflinger G., Frei R., Kristiansen K., Frei K.M., 2020. A strontium isotope baseline of Cyprus. Assessing the use of soil leachates, plants, groundwater and surface water as proxies for the local range of bioavailable strontium isotope composition. Science of Total Environment. 15, 708:134714. doi: 10.1016/j.scitotenv.2019.134714 31787293

[41] Scaffidi B.K., Knudson K.J., 2020. An archaeological strontium isoscape for the prehistoric Andes: understanding population mobility through a geostatistical meta-analysis of archaeological ^87^Sr/^86^Sr values from humans, animals, and artifacts. Journal of Archaeological Science. 117, 105121.

[42] Snoeck et al. 2020. Towards a biologically available strontium isotope baseline for Ireland. Science of Total Environment. 712, 136248. doi: 10.1016/j.scitotenv.2019.136248 31945525

[43] Wang X., Tang Z., 2020. The first large-scale bioavailable Sr isotope map of China and its implication for provenance studies. Earth-Science Reviews. 103353.

[44] Frank A. B., Frei R., Triantaphyllou M., Vassilakis E., Kristiansen K., Frei K. M., 2021. Isotopic range of bioavailable strontium on the Peloponnese peninsula, Greece: A multi-proxy approach. Science of the Total Environment. 774, 145181.

[45] Funck J., Bataille C., Rasic J., Wooller M., 2021. A bio‐available strontium isoscape for eastern Beringia: a tool for tracking landscape use of Pleistocene megafauna. Journal of Quaternary Science. 36(1), 76–90.

[46] Washburn E., Nesbitt J., Ibarra B., Fehren-Schimtz L., Oelze V.M., 2021. A strontium isoscape for the Conchucos region of highland Perù and its application to Andean archaeology. PloS One. 16, e0248209. 10.1371/journal.pone.0248209 PMC8009355 33784347

[47] Zieliński M., Dopieralska J., Królikowska-Ciągło S., Walczak A., Belka Z., 2021. Mapping of spatial variations in Sr isotope signatures (87Sr/86Sr) in Poland—Implications of anthropogenic Sr contamination for archaeological provenance and migration research. Science of Total Environment. 775, 145792. 10.1016/j.scitotenv.2021.145792 33631577

[48] Kootker L.M., van Lanen R.J., Kars H., Davies G.R., 2016. Strontium isoscape in the Netherlands: spatial variations in ^87^Sr/^86^Sr as a proxy of palaeomobility. Journal of Archaeological Science: Reports. 6, 1–13.

[49] Evans J.A., Mee K., Chenery C.A., Cartwright C.E., Lee K.A., Marchant A.P., 2018. User guide for the Biosphere Isotope Domains GB (Version 1) dataset and web portal. British Geological Survey Open Report, OR/18/005, 21.

[50] Barbarena et al. 2017. Scale of human mobility in the southern Andes (Argentina and Chile): a new framework based on strontium isotopes. American Journal of Physical Anthropology. 164, 305–320. doi: 10.1002/ajpa.23270 28631376

[51] Emery M.V., Stark R.J., Murchie T.J., Elford S., Schwarcz H.P., Prowse T.L., 2018. Mapping the origins of Imperial Roman workers (1st-4th century CE) at Vagnari, Southern Italy, using 87Sr/86Sr and δ18O variability. American Journal of Physical Anthropology. 16, 837–850. 10.1002/ajpa.23473 29667172

[52] Lugli F., Cipriani A., Bruno L., Ronchetti F., Cavazzuti C., Benazzi S., 2022. A strontium isoscape of Italy for prevenance studies. Chemical Geology. 587, 1–10.

[53] Wunder M. B. (2010). Using isoscapes to model probability surfaces for determining geographic origins. Isoscapes: understanding movement, pattern, and process on Earth through isotope mapping: 251–270.

[54] Carmignani L., Oggiano G., Funedda A., Conti P., Pasci S., 2016. The geological map of Sardinia (Italy) at 1: 250,000 scale. Journal of Maps. 12(5), 826–835

[55] Lugliè C., 2018. Your Path Led Trough the Sea… The Emergence of Neolithic in Sardinia and Corsica. Quaternary International. 470, 285–300.

[56] Tykot R.H., 1996. Obsidian Procurement and Distribution in the Central and Western Mediterranean. Journal of Mediterranean Archaeology. 9, 39–82.

[57] Freund K.P., Batist Z., 2014. Sardinian obsidian circulation and early maritime navigation in the Neolithic as shown through social network analysis. Journal of Island and Coastal Archaeology. 9(3), 364–380.

[58] Tykot R.H., 2021. Non-Destructive Pxrf on Prehistoric Obsidian Artifacts from the Central Mediterranean. Applied Sciences. 11:16, 7459.

[59] Bernardini, P., 2011a. Le torri, i metalli, il mare. Storie antiche di un’isola mediterranea. Sassari.

[60] Holt E., 2015. Mobility and meaning in the Nuragic Culture of Bronze Age Sardinia (ca. 1700–900 BC). Chemical Geology. 232, 54–66.

[61] Freund K.P., 2018. Lunati and the island of the towers: obsidian in Nuragic Sardinia. Journal of Archaeological Science: Reports. 21, 1–9.

[62] Vagnetti L., Lo Schiavo F., 1989. Late Bronze Age long-distance trade: the role of the Cypriots. Early Society in Cyprus. 217–243.

[63] Balmuth M.S., 1992. Archaeology in Sardinia. American Journal of Archaeology. 96(4), 663–697.

[64] Santoni, V., 2001. Il Nuraghe Su Nuraxi Di Barumini. Ministero per i Beni e le Attività Culturali, Soprintendenza Archeologica per le Province di Cagliari e Oristano.

[65] Moscati S., Bartoloni P., Bondì S.F., 1997. La penetrazione fenicia e punica in Sardegna trent’anni dopo, Accademia nazionale dei Lincei. Classe di scienze morali, storiche e filologiche. Memorie. Roma

[66] van Dommelen P., 1997. Colonial constructs: colonialism and archaeology in the Mediterranean. World Archaeology. 28, 305–23.

[67] Guirguis M., Murgia C., Pla Orquín R., 2017. From The Mediterranean to The Atlantic: People, Goods and Ideas Between East and West. Proceedings of the 8th International Congress of Phoenician and Punic Studies (Italy, Sardinia-Carbonia, Sant’antioco, 21st–26th of October 2013), Folia Phoenicia. 1, 282–299.

[68] Marcus J.H., Posth C., Ringbauer H. et al. 2020. Genetic history from the Middle Neolithic to present on the Mediterranean island of Sardinia. Nature Communications. 11 (1), 939. doi: 10.1038/s41467-020-14523-6 32094358 PMC7039977

[69] Bartoloni P., 1995. Le line commerciali all’alba del primo millennio. In: Moscati S. (Ed), I Fenici: ieri, oggi, domani. Ricerche, scoperte, progetti, Accademia Nazionale dei Lincei, Roma, pp. 245–259.

[70] Aubet M.E., 2001. The Phoenician and the West. Politics, Colonies, and Trade, second ed. Cambridge University Press, New York.

[71] Guirguis M., (2019). Central North Africa and Sardinian connections (end of 9th-8th century BC). The multi-ethnic and multicultural facies of the earliest western Phoenician communities. Archaeology in Africa, 111–125.

[72] Delgado A., Ferrer M., 2007. Cultural Contacts in Colonial Settings: The Construction of New Identities in Phoenician Settlements of the Western Mediterranean. Stanford Journal of Archaeology. 5, 18–42.

[73] Parrot, A.,Chéhab, M.H., Moscati, S., 1982. I Fenici. L’espansione fenicia. Cartagine. Milano.

[74] Bartoloni P., 1979. L’antico porto di Nora. Antiqua (13), 57–61.

[75] Finocchi S., 1999. La laguna e l’antico porto di Nora: nuovi dati a confronto. Rivista di Studi Fenici. XXVII, 2, 167–192.

[76] Bonetto J., 2009. L’insediamento di età fenicia, punica e romana repubblicana nell’area del foro. In: Bonetto J., Ghiotto A.R., Novello M., (Eds.), Nora. Il foro romano. Storia di un’area urbana dall’età fenicia alla tarda antichità (1997–2006). I. Lo scavo, Scavi di Nora I, Padova, pp. 39–243.

[77] Tronchetti C., 2010. La facies fenicia di Nora. Rivista di Studi Fenici. XXXVIII, 119–130.

[78] Bonetto J., Marinello A., Zara A., 2021. Nuovi dati di scavo e vecchi documenti d’archivio. Il santuario di Esculapio e le più antiche presenze a Nora. In: Bettineschi C., Burigana L., Magnini L., (Eds.), Trace of Complexity. Studi in onore di Armando De Guio, Mantova, pp. 193–222.

[79] Bonetto J., 2014. L’insediamento fenicio di Nora e le comunità nuragiche circostanti: contatti e distanze. In: van Dommelen P., Roppa A., (Eds.), Materiali e contesti nell’Età del Ferro sarda. Atti della giornata di studi (Museo Civico di San Vero Milis, Oristano, 25 maggio 2012), Rivista di Studi Fenici, XLI, pp. 173–182.

[80] Bernardini P., 2011b. Dalla stele di Nora agli scavi nel foro: i fenici ritrovati. In: Bonetto J., Falezza G., (Eds.), Vent’anni di scavi a Nora. Ricerca, formazione e politica culturale. 1990–2009, Scavi di Nora II, Roma, pp. 127–136.

[81] Bonetto J., 2021. Nora fenicia. Nuovi dati e nuove letture. In: Bondì S.F., Botto M., Garbati G., Oggiano I., (Eds.), Tra le coste del Levante e le terre del tramonto. Studi in ricordo di Paolo Bernardini, Collezione di Studi Fenici, 51, Roma, pp. 195–208.

[82] Mazzariol A., 2021. La tomba T36 della necropoli della necropoli occidentale di Nora. Sardinia, Corsica et Baleares Antiquae: An International Journal of Archaeology. XIX, 93–128.

[83] Bonetto et al. 2022. La necropoli fenicia e punica di Nora: saggi 1 e 4. Indagini 2021. Quaderni Norensi. 9, 241–272.

[84] Bonetto J., Botto M., 2017. Tra i primi a Nora. Una sepoltura a cremazione nella necropoli sull’istmo. Quaderni. Rivista di Archeologia. Soprintendenza Archeologia, Belle Arti e Paesaggio per la città metropolitana di Cagliari e le province di Oristano e Sud Sardegna. 28, 193–214.

[85] Carmignani et al. 2001. Geologia della Sardegna, Servizio Geologico d’Italia. 272.

[86] Thomsen E., Andreasen R., 2019. Agricultural lime disturbs natural strontium isotope variations: implication for provenance and migration studies. Science Advances. 5, 1–11. 10.1126/sciadv.aav8083 PMC6415960 30891501

[87] Bonetto et al. 2020. La necropoli fenicia e punica occidentale: le indagini 2018–2019. Quaderni Norensi. 8, 187–215.

[88] Bandera S., Tecchiati U., 2021. I reperti osteologici. In: Bonetto J., Mantovani V., Zara A., (Eds). Nora. Il Tempio romano 2008–2014. II.2. I materiali e gli altri reperti. Roma, pp. 573–610.

[89] Jung M. J., Yim S. G., Jeong Y. J., Jeong G. Y., Ryu J. S., & Cheong A. C. S. (2020). Characterization of bioavailable Sr isotopic composition of Jeju Island, Korea. Geosciences Journal, 24, 625–632.

[90] Argentino C., Lugli F., Cipriani A., & Panieri G. (2021). Testing miniaturized extraction chromatography protocols for combined 87Sr/86Sr and δ88/86Sr analyses of pore water by MC‐ICP‐MS. Limnology and Oceanography. Methods. 19(6), 431–440.

[91] Lugli F., Cipriani A., Arnaud J., Arzarello M., Peretto C., & Benazzi S. (2017). Suspected limited mobility of a Middle Pleistocene woman from Southern Italy: strontium isotopes of a human deciduous tooth. Scientific Reports. 7(1), 8615. doi: 10.1038/s41598-017-09007-5 28819227 PMC5561174

[92] Sighinolfi G. P., Lugli F., Piccione F., Michele V. D., Cipriani A., 2020. Terrestrial target and melting site of Libyan Desert Glass: New evidence from trace elements and Sr isotopes. Meteoritics & Planetary Science. 55, 1865–1883.

[93] Lugli F., Cipriani A., Peretto C., Mazzucchelli M., Brunelli D., 2017. In situ high spatial resolution 87Sr/86Sr ratio determination of two Middle Pleistocene (ca 580 ka) Stephanorhinus hundsheimensis teeth by LA–MC–ICP–MS. International Journal of Mass Spectrometry. 412, 38–48.

[94] Lugli F., Cipriani A., Tavaglione V., Traversari M., Benazzi S., 2018. Transhumance pastoralism of Roccapelago (Modena, Italy) early‐modern individuals: Inferences from Sr isotopes of hair strands. American Journal of Physical Anthropology. 167(3), 470–483. doi: 10.1002/ajpa.23643 30159877

[95] Berglund M., Wieser M.E., 2011. Isotopic compositions of the elements 2009 (IUPAC Technical Report). Pure and applied chemistry. 83(2), 397–410.

[96] Maurer A. F., Galer S. J., Knipper C., Beierlein L., Nunn E. V., Peters D., Tütken T., Alt K. W., Schöne B. R. (2012). Bioavailable 87Sr/86Sr in different environmental samples—Effects of anthropogenic contamination and implications for isoscapes in past migration studies. Science of the Total Environment. 433, 216–229. doi: 10.1016/j.scitotenv.2012.06.046 22796412

[97] Bataille C.P., Crowley B.E., Wooller M.J., Bowen G.J., 2020. Advances in global bioavailable strontium isoscape. Palaeogeography, Palaeoclimatology, Palaeoecology. 555, 109849.

[98] Bataille C.P., Brennan S.R., Hartmann J., Moosdorf N., Wooller M.J., Bowen G.J., 2014. A geostatistical framework for predicting variations in strontium concentrations and isotope ratios in Alaskan rivers. Chemical Geology. 389, 1–15.

[99] Hartmann J., Moosdorf N., 2012. The new global lithological map database GLiM: A representation of rock properties at the Earth surface. Geochemistry, Geophysics, Geosystems. 13(12), 1–26.

[100] Ma C., Vander Zanden H.B., Wunder M.B., Bowen G.J., 2020. assignR: an R package for isotope‐based geographic assignment. Methods in Ecology and Evolution. 11(8), 996–1001.

[101] Alonzi E., Pacheco-Forés S.I., Gordon G.W., Kuijit I., Knudson K.J, 2020. New understandings of the sea spray effect and its impact on bioavailable radiogenic strontium isotope ratios in coastal environments. Journal of Archaeological Science: Reports. 33, 102462.

[102] Botto M., 2011. 1992–2022: dieci anni di prospezioni topografiche a Nora e nel suo territorio. In: Bonetto J., Falezza G., (Eds.), Vent’anni di scavi a Nora. Ricerca, formazione e politica culturale. 1990–2009, Scavi di Nora II, Roma, pp. 56–84.

[103] Finocchi S., 2013. Dalla Nora fenicia alla Nora punica e oltre. Le sette città di Nora. Atti della giornata (Milano, 11 febbraio 2013). 157–179.

[104] Puddu G., Maiorano L., Falcucci A., Corsi F., Boitani L., 2009. Spatial-explicit assessment of current and future conservation options for the endangered Corsican Red Deer (Cervus elaphus corsicanus) in Sardinia. Biodiversity and conservation. 18(8), 2001–2016.

[105] Lombardini M., Meriggi A., Fozzi A., 2017. Factors influencing wild boar damage to agricultural crops in Sardinia (Italy). Current Zoology. 63(5), 507–514. doi: 10.1093/cz/zow099 29492010 PMC5804203

