# Supplementary figures and images for: Machine learning-based Sr isoscape of southern Sardinia: A tool for bio-geographic studies at the Phoenician-Punic site of Nora
Source: PLoS One. 2023 Jul 19;18(7):e0287787. doi: 10.1371/journal.pone.0287787 (PMC10355458; doi:10.1371/journal.pone.0287787)

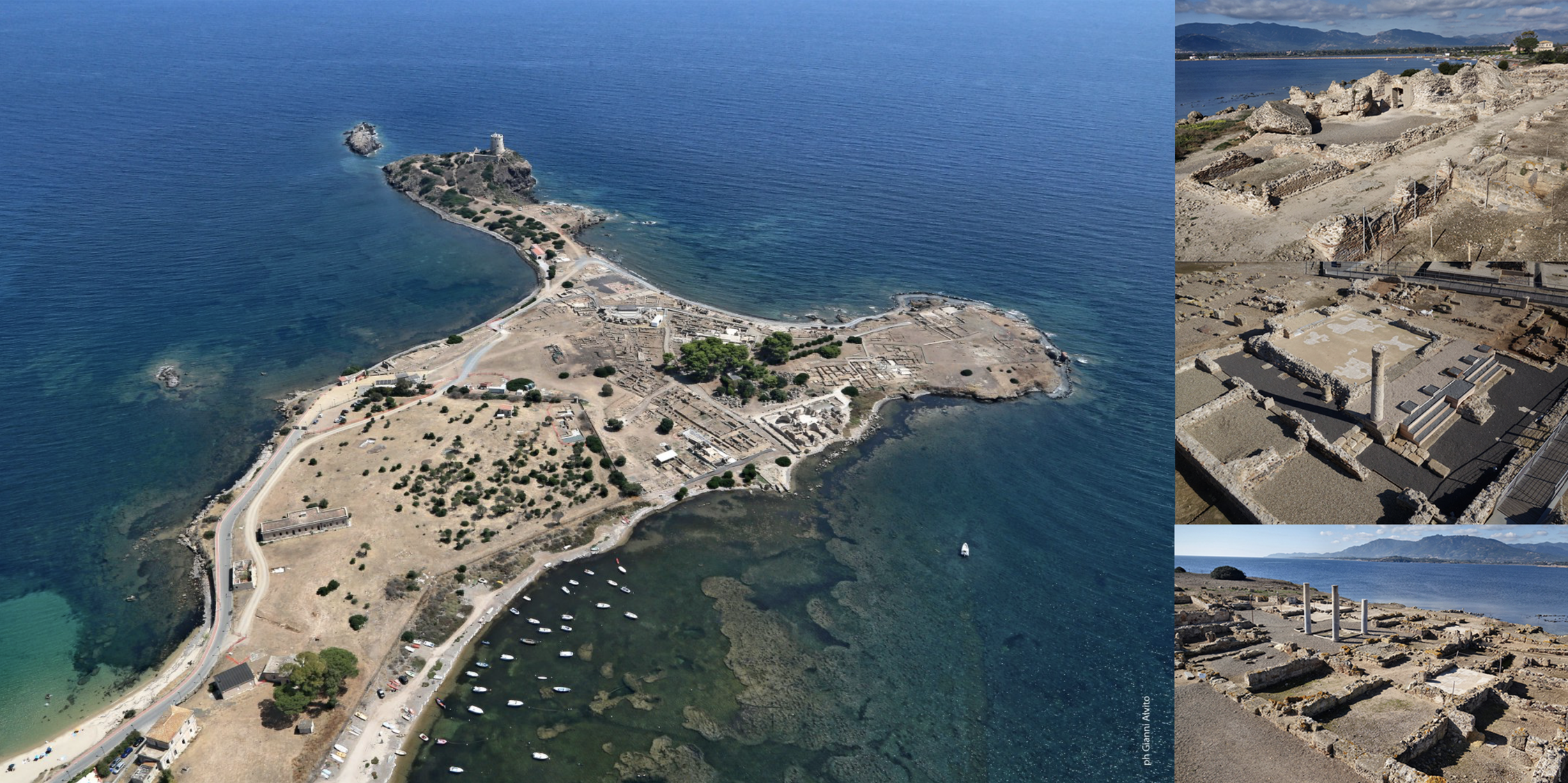

Supplement: S1 Fig — On the left is the archaeological site of Nora. On the right are some ancient monuments of the Imperial Roman period (a., Terme a mare–Bath by the sea; b., Tempio Romano–Roman Temple; c., Casa dell’Atrio tetrastilo–House of the Tetrasyle Atrium. These images are the propriety of the Department of Cultural Heritage–at the University of Padua. The photographer Gianni Alvito (Teravista, CA) provided us with courtesy of the Ministero della Cultura–Italian Ministry for Culture (former Ministero per i Beni e le Attività Culturali e il Turismo), Soprintendenza Archeologia, Belle Arti e Paesaggio per l’area metropolitana di Cagliari e le province di Oristano e Sud Sardegna. (TIF) [file pone.0287787.s001.tif]
