# Supplementary material for: Machine learning-based Sr isoscape of southern Sardinia: A tool for bio-geographic studies at the Phoenician-Punic site of Nora
Source: PLoS One. 2023 Jul 19;18(7):e0287787. doi: 10.1371/journal.pone.0287787 (PMC10355458; doi:10.1371/journal.pone.0287787)
Supplement: S1 Table — List of the environmental samples analysed in this work and the 87Sr/86Sr isotope ratio results for each sample. (DOCX) [file pone.0287787.s004.docx]

| \| **Supplementary Table 1. *Sr isotope results of the samples considered in this study***  List of the environmental samples analysed in this work and the ^87^Sr/^86^Sr isotope ratio results for each sample. \| \| \| \| \| \| \| \| \| --- \| --- \| --- \| --- \| --- \| --- \| --- \| --- \| \| Sampling site \| Latitude \| Longitude \| Lab ID \| Sample type \| Sample characteristics \| ^87^Sr/^86^Sr \| 2 SE \| \| Nr Aesculapius \| 38.9846 \| 9.0168 \| BASR_01 \| rock \| andesite \| 0.70702 \| 0.00001 \| \| Nr Aesculapius \| 38.9846 \| 9.0168 \| BASR_02 \| plant \| - \| 0.70878 \| 0.00001 \| \| Nr Coltellazzo \| 38.9846 \| 9.0168 \| BASR_03 \| soil \| soil (-20 cm) \| 0.70933 \| 0.00001 \| \| Nr Coltellazzo \| 38.9846 \| 9.0168 \| BASR_04 \| plant \| - \| 0.70933 \| 0.00001 \| \| Nr Est Forum \| 38.9846 \| 9.0168 \| BASR_05 \| water \| groundwater \| 0.70896 \| 0.00002 \| \| Nr Roman Temple \| 38.9846 \| 9.0168 \| BASR_06 \| soil \| soil (-20 cm) \| 0.70957 \| 0.00001 \| \| Nr Roman Temple \| 38.9846 \| 9.0168 \| BASR_07 \| soil \| soil (Punic level) \| 0.70930 \| 0.00001 \| \| Nr Roman Temple \| 38.9846 \| 9.0168 \| BASR_08 \| mammal \| A-ENML sheep \| 0.70899 \| 0.00001 \| \| Nr Central Thermae \| 38.9846 \| 9.0168 \| BASR_09 \| water \| meteoric water \| 0.70902 \| 0.00001 \| \| Nr Pozzo nuragico \| 38.9846 \| 9.0168 \| BASR_10 \| water \| groundwater \| 0.70888 \| 0.00001 \| \| Nr Tanit Hill \| 38.9846 \| 9.0168 \| BASR_11 \| rock \| andesite \| 0.70763 \| 0.00001 \| \| Nr Tanit Hill \| 38.9846 \| 9.0168 \| BASR_12 \| plant \| - \| 0.70969 \| 0.00001 \| \| Nr Necropolis N-W \| 38.9846 \| 9.0168 \| BASR_13 \| rock \| sandstone \| 0.71026 \| 0.00001 \| \| Nr Necropolis N-W \| 38.9846 \| 9.0168 \| BASR_14 \| plant \| - \| 0.70950 \| 0.00001 \| \| Nr Necropolis N-W \| 38.9846 \| 9.0168 \| BASR_15 \| soil \| soil (burial) \| 0.70952 \| 0.00001 \| \| Nr Necropolis N-W \| 38.9846 \| 9.0168 \| BASR_16 \| mammal \| A-ENML sheep \| 0.70824 \| 0.00001 \| \| Pula plain \| NA \| NA \| BASR_17 \| mammal \| M-ENML sheep \| 0.71060 \| 0.00001 \| \| Pula plain \| NA \| NA \| BASR_18 \| mammal \| M-ENML deer \| 0.71106 \| 0.00001 \| \| Pula plain \| NA \| NA \| BASR_19 \| mammal \| M-ENML boar \| 0.70958 \| 0.00001 \| \| Pula plain \| NA \| NA \| BASR_20 \| mammal \| M-BN deer \| 0.71021 \| 0.00001 \| \| Zone 1 \| 38.9599 \| 8.9597 \| BASR_21 \| rock \| granite \| 0.85613 \| 0.00002 \| \| Zone 1 \| 38.9599 \| 8.9597 \| BASR_22 \| plant \| - \| 0.71035 \| 0.00001 \| \| Zone 1 \| 38.9599 \| 8.9597 \| BASR_23 \| soil \| soil (-20 cm) \| 0.71019 \| 0.00001 \| \| Zone 2 \| 38.9773 \| 8.9599 \| BASR_24 \| plant \| - \| 0.71179 \| 0.00001 \| \| Zone 2 \| 38.9773 \| 8.9599 \| BASR_25 \| soil \| soil (-20 cm) \| 0.71173 \| 0.00001 \| \| Zone 3 \| 38.9907 \| 8.9661 \| BASR_26 \| plant \| - \| 0.70831 \| 0.00001 \| \| Zone 3 \| 38.9907 \| 8.9661 \| BASR_27 \| soil \| soil (-20 cm) \| 0.70852 \| 0.00001 \| \| Zone 4 \| 39.0099 \| 8.9687 \| BASR_28 \| plant \| - \| 0.70872 \| 0.00001 \| \| Zone 4 \| 39.0099 \| 8.9687 \| BASR_29 \| soil \| soil (-20 cm) \| 0.70827 \| 0.00001 \| \| Zone 5 \| 39.0231 \| 8.9802 \| BASR_30 \| plant \| - \| 0.71005 \| 0.00001 \| \| Zone 5 \| 39.0231 \| 8.9802 \| BASR_31 \| soil \| soil (-20 cm) \| 0.71031 \| 0.00001 \| \| Zone 6 \| 38.9681 \| 8.9736 \| BASR_32 \| plant \| - \| 0.71070 \| 0.00001 \| \| Zone 7 \| 38.9179 \| 8.8407 \| BASR_33 \| plant \| - \| 0.71294 \| 0.00001 \| \| Zone 8 \| 39.0126 \| 8.8816 \| BASR_34 \| plant \| - \| 0.71040 \| 0.00001 \| \| Zone 9 \| 38.8981 \| 8.8885 \| BASR_35 \| plant \| - \| 0.71020 \| 0.00001 \| \| Zone 10 \| 38.9042 \| 8.8913 \| BASR_36 \| plant \| - \| 0.71091 \| 0.00001 \| \| Zone 11 \| 39.0606 \| 9.0362 \| BASR_37 \| plant \| - \| 0.70900 \| 0.00002 \| \| Zone 12 \| 39.0680 \| 8.9849 \| BASR_38 \| plant \| - \| 0.70879 \| 0.00001 \| \| Zone 13 \| 39.2553 \| 8.9935 \| BASR_39 \| plant \| - \| 0.71125 \| 0.00001 \| \| Zone 14 \| 39.1923 \| 9.1565 \| BASR_40 \| plant \| - \| 0.70937 \| 0.00001 \| \| Zone 15 \| 39.2024 \| 9.4676 \| BASR_41 \| plant \| - \| 0.71251 \| 0.00001 \| \| Zone 16 \| 39.3226 \| 9.4011 \| BASR_42 \| plant \| - \| 0.71174 \| 0.00001 \| \| Zone 17 \| 39.5136 \| 9.2713 \| BASR_43 \| plant \| - \| 0.70966 \| 0.00001 \| \| Zone 18 \| 39.5573 \| 9.2529 \| BASR_44 \| plant \| - \| 0.71165 \| 0.00001 \| \| Zone 19 \| 39.4616 \| 9.1609 \| BASR_45 \| plant \| - \| 0.70995 \| 0.00001 \| \| Zone 20 \| 39.3843 \| 9.0756 \| BASR_46 \| plant \| - \| 0.71016 \| 0.00001 \| \| Zone 21 \| 38.9599 \| 8.7630 \| BASR_47 \| plant \| - \| 0.71038 \| 0.00001 \| \| Zone 22 \| 38.9423 \| 8.7474 \| BASR_48 \| plant \| - \| 0.71157 \| 0.00001 \| \| Zone 23 \| 38.9315 \| 8.7119 \| BASR_49 \| plant \| - \| 0.71107 \| 0.00001 \| \| Zone 24 \| 39.0084 \| 8.6481 \| BASR_50 \| plant \| - \| 0.71001 \| 0.00001 \| \| Zone 25 \| 38.9898 \| 8.6270 \| BASR_51 \| plant \| - \| 0.70971 \| 0.00001 \| \| Zone 26 \| 38.9695 \| 8.5936 \| BASR_52 \| plant \| - \| 0.70921 \| 0.00001 \| \| *Nr = Nora (archaeological site); Necropolis N-W = north-western necropolis of Nora; A-ENML = ancient enamel sample; M-ENML = modern enamel sample; M-BN = modern bone sample; NA = coordinates not available. \| \| \| \| \| \| \| \| |
| --- | --- | --- | --- | --- | --- | --- | --- | --- | --- | --- | --- | --- | --- | --- | --- | --- | --- | --- | --- | --- | --- | --- | --- | --- | --- | --- | --- | --- | --- | --- | --- | --- | --- | --- | --- | --- | --- | --- | --- | --- | --- | --- | --- | --- | --- | --- | --- | --- | --- | --- | --- | --- | --- | --- | --- | --- | --- | --- | --- | --- | --- | --- | --- | --- | --- | --- | --- | --- | --- | --- | --- | --- | --- | --- | --- | --- | --- | --- | --- | --- | --- | --- | --- | --- | --- | --- | --- | --- | --- | --- | --- | --- | --- | --- | --- | --- | --- | --- | --- | --- | --- | --- | --- | --- | --- | --- | --- | --- | --- | --- | --- | --- | --- | --- | --- | --- | --- | --- | --- | --- | --- | --- | --- | --- | --- | --- | --- | --- | --- | --- | --- | --- | --- | --- | --- | --- | --- | --- | --- | --- | --- | --- | --- | --- | --- | --- | --- | --- | --- | --- | --- | --- | --- | --- | --- | --- | --- | --- | --- | --- | --- | --- | --- | --- | --- | --- | --- | --- | --- | --- | --- | --- | --- | --- | --- | --- | --- | --- | --- | --- | --- | --- | --- | --- | --- | --- | --- | --- | --- | --- | --- | --- | --- | --- | --- | --- | --- | --- | --- | --- | --- | --- | --- | --- | --- | --- | --- | --- | --- | --- | --- | --- | --- | --- | --- | --- | --- | --- | --- | --- | --- | --- | --- | --- | --- | --- | --- | --- | --- | --- | --- | --- | --- | --- | --- | --- | --- | --- | --- | --- | --- | --- | --- | --- | --- | --- | --- | --- | --- | --- | --- | --- | --- | --- | --- | --- | --- | --- | --- | --- | --- | --- | --- | --- | --- | --- | --- | --- | --- | --- | --- | --- | --- | --- | --- | --- | --- | --- | --- | --- | --- | --- | --- | --- | --- | --- | --- | --- | --- | --- | --- | --- | --- | --- | --- | --- | --- | --- | --- | --- | --- | --- | --- | --- | --- | --- | --- | --- | --- | --- | --- | --- | --- | --- | --- | --- | --- | --- | --- | --- | --- | --- | --- | --- | --- | --- | --- | --- | --- | --- | --- | --- | --- | --- | --- | --- | --- | --- | --- | --- | --- | --- | --- | --- | --- | --- | --- | --- | --- | --- | --- | --- | --- | --- | --- | --- | --- | --- | --- | --- | --- | --- | --- | --- | --- | --- | --- | --- | --- | --- | --- | --- | --- | --- | --- | --- | --- | --- | --- | --- | --- | --- | --- | --- | --- | --- | --- | --- | --- | --- | --- | --- | --- | --- | --- | --- | --- | --- | --- | --- | --- | --- | --- | --- | --- | --- | --- | --- | --- | --- | --- | --- | --- | --- | --- | --- | --- | --- | --- | --- | --- | --- | --- | --- | --- | --- | --- | --- | --- | --- | --- | --- | --- | --- | --- | --- | --- | --- | --- | --- |
