# Supplementary material for: Machine learning-based Sr isoscape of southern Sardinia: A tool for bio-geographic studies at the Phoenician-Punic site of Nora
Source: PLoS One. 2023 Jul 19;18(7):e0287787. doi: 10.1371/journal.pone.0287787 (PMC10355458; doi:10.1371/journal.pone.0287787)
Supplement: S1 File — (DOCX) [file pone.0287787.s005.docx]

**Additional Information**

The current research was accomplished following the relevant regulations outlined in the International Council of Archaeozoology (ICAZ) for the treatment and the destructive sampling of archaeofauna material. Furthermore, the gathering of teeth and bone samples from modern deer and boar complied with the Berne Convention on the Conservation of European Wildlife and Natural Habits (Council Decision 82/72/EEC; OJ L 38, 10.2.1982). The research activities at the ancient site of Nora were conducted under the Research Concession by the Ministero della Cultura – Italian Ministry for Culture (former Ministero per i Beni e le Attività Culturali e il Turismo) by Decree 916 (30.08.2019).

Sampling selection and cutting process of both archaeological and modern remains were performed at the Laboratory of Archaeology – Department of Cultural Heritage of the University of Padua; whilst isotope analysis was carried out at the Department of Chemical and Geological Sciences and the Centro Interdipartimentale Grandi Strumenti (CIGS) of the University of Modena & Reggio Emilia.

As an additional note to the CReditT author statement (Contributor Roles Taxonomy), in accordance with the Consiglio Universitario Nazionale – Italian National University Council – of the Ministero dell’Università e della Ricerca (MIUR) for evaluating the scientific production in the disciplinary field of Antiquity, Philology, Literary Studies, Art History (L-ANT/01 – L-ANT/10), we must specify that A.M. wrote Section 1.1.1. *‘The Phoenician and Punic site of Nora’*.
